# Distribution of endotoxin in maternal and fetal body with intrahepatic cholestasis of pregnancy and its association with adverse fetal outcome

**DOI:** 10.1186/s12884-022-05235-4

**Published:** 2022-12-08

**Authors:** Xiaomei Huang, Lei Lei, Fan Feng, Yong Shao

**Affiliations:** 1 Department of Obstetrics and Gynecology, The First Affiliated Hospital of Chongqing Medical University, Chongqing, China; 2 Department of Obstetrics and Gynecology, The People’s Hospital of Kaizhou District, Chongqing, China; 3 Depatement of Obstetrics and Gynecology, Chongqing Jiangbei District Maternal and Child Health Hospital, Chongqing, China

**Keywords:** Endotoxin, Lipopolysaccharide, Intrahepatic cholestasis of pregnancy, Ursodeoxycholic acid, Resveratrol

## Abstract

**Background:**

Intrahepatic cholestasis of pregnancy is a pregnancy-specific liver disease. In this study, we sought to explore the distribution of lipopolysaccharide in the maternal body, and its effect on the fetal body in the intrahepatic cholestasis of pregnancy mice. It provides a new sight for the clinical treatment of women with intrahepatic cholestasis of pregnancy.

**Methods:**

The serum levels of lipopolysaccharide and lipopolysaccharide binding protein in women with intrahepatic cholestasis of pregnancy were analyzed. To assess the association between lipopolysaccharide levels and adverse fetal outcomes, ursodeoxycholic acid, resveratrol, and phosphatidylinositol-3-kinase inhibitor were employed in intrahepatic cholestasis of pregnancy mice, and we studied the fluorescence intensity and distribution of lipopolysaccharide in mice with intrahepatic cholestasis of pregnancy.

**Results:**

Our data indicated significantly elevated levels of lipopolysaccharide and lipopolysaccharide binding protein in women with intrahepatic cholestasis of pregnancy. In vivo fluorescence imaging revealed that the intensity of lipopolysaccharide in mice with intrahepatic cholestasis of pregnancy was higher than that in the control group, and decreased after ursodeoxycholic and resveratrol treatment. The fluorescence intensity analysis indicated that lipopolysaccharide levels in maternal liver, placenta, fetal brain and fetal liver were significantly higher in the intrahepatic cholestasis pregnancy mice group than in the control group.

**Conclusions:**

This study provided evidence of endotoxin distribution in maternal liver, placenta, fetal liver and fetal brain in mice with intrahepatic cholestasis of pregnancy. Ursodeoxycholic acid and resveratrol treatment effectively reduced lipopolysaccharide levels in pregnant mice with intrahepatic cholestasis of pregnancy.

**Supplementary Information:**

The online version contains supplementary material available at 10.1186/s12884-022-05235-4.

## Background

Intrahepatic cholestasis of pregnancy (ICP) is a pregnancy-specific disease that occurs in the middle and late pregnancy and resolves rapidly post delivery. ICP is mainly characterized by maternal skin itching and bile acid elevation [1, 2]. While the pathogenesis of ICP is multifactorial, direct evidence indicated a stronger association between ICP and adverse perinatal outcomes, such as the increased risk for prematurity, intrapartum fetal distress, and stillbirth. Therefore, identifying the possible mechanism of ICP-induced poor fetal outcome may explain this phenomenon, and is helpful in the development of the clinical intervention.

The gastrointestinal mucosal epithelium is a semipermeable barrier that allows the absorption of nutrients, while restricting harmful microorganisms and molecules into the systemic circulation. Increased intestinal permeability is usually observed in inflammatory bowel disease and intestinal infections [3]. Gut microbiota dysbiosis, characterized by an imbalance in the composition and activity of gut microbial communities, has been recently suggested to be involved in intestinal barrier dysfunction, and contributed to the establishment of metabolic diseases such as obesity [4], type 2 diabetes (T2D) [5], inflammatory bowel disease (IBD) [6], and possibly ICP [7, 8]. Li et al. [9] reported that the intestinal tract of women with ICP was rich in Bilophila, Parabacteroides, and Shigella, but lacking in Faecalibacterium. Imbalanced composition and activity of gut microbial communities might increase intestinal permeability, and facilitate endotoxin influx into the systemic circulation. HU et al. [10] demonstrated that the serum endotoxin level of women with ICP was significantly higher than that of normal pregnant women. Humberto et al. [8] found that the level of anti-LPS IgM antibody in the serum of women with ICP was higher than in normal pregnant women.

Lipopolysaccharide (LPS) is the main component of endotoxin with various biological activities. LPS combines into lipopolysaccharide binding protein (LBP) in monocytes, then promotes the production of inflammatory factors [11, 12]. Beom etc. [11, 13] have shown that LPS in the blood bind to LBP and act on CD14 to form toll-like receptor 4 (TLR4)-MD-2 complex, which activates the NF-kB pathway and produces a large number of inflammatory factors including tumor necrosis factor α(TNF-α). As a proinflammatory cytokine, TNF-α stimulated oxygen free radicals production in neutrophils, and further activated inflammatory cytokines release in macrophages, triggering an inflammatory cascade reaction. During pregnancy, exposure to LPS is associated with delayed neonatal arousal, systemic inflammation, altered serum biochemistry, and increased urinary protein and sodium excretion in fetal and newborns [14].

In the present study, we sought to explore the effect and distribution of LPS in the maternal body and its effect on the fetal body. The serum levels of LPS and LBP in women with ICP were analyzed, and the distribution of LPS was detected in ICP mice and embryos. To assess the association between LPS and adverse fetal outcomes, ursodeoxycholic (UDCA), resveratrol (RES), and phosphatidylinositol-3-kinase (PI3K) inhibitor (LY294002) were employed in groups.

## Methods

### Study design

The clinical study samples in this paper are the serum of pregnant women from the Department of Obstetrics and Gynecology of the First Affiliated Hospital of Chongqing Medical University from 2021 to 2022, which is a cross-sectional study. The pregnant mice used in this study were obtained from Chengdu Dashuo Company. All patients signed informed consent. The present study was approved by the ethical committee of the First Affiliated Hospital of Chongqing Medical University (Ethical Application Ref: 2021–533).

### Human blood samples

Blood samples were collected from 83 cases of pregnant women who underwent cesarean section in the First Affiliated Hospital of Chongqing Medical University from 2021 to 2022 (including 20 cases of normal pregnant women, 19 cases of ICP subgroup 1, 23 cases of ICP subgroup 2 and 21 cases of ICP subgroup 3). According to the 5.5% incidence of ICP in Chongqing, China, the sample size was about 18. 2476 calculated by the formula ($\textrm{n}=\frac{{\textrm{Z}}^2\times \textrm{p}\times \left(1-\textrm{p}\right)}{\updelta^2}$), so we selected 20 samples as the normal group. According to the different concentration of total bile acid (TBA) and alanine aminotransferase (ALT), subjects were divided into ICP subgroup 1 (TBA > 10 μmol/L, ALT> 40 U/L); ICP subgroup 2 (TBA > 10 μmol/L, ALT< 40 U/L); ICP subgroup 3 (TBA < 10 μmol/L, ALT> 40 U/L). The ICP was included according to the internationally accepted ICP diagnostic criteria [15, 16]. Exclusion criteria for ICP subgroups were abnormalities in liver function and bile metabolism before pregnancy, gestational diabetes mellitus, hypertensive disorder complicating pregnancy, premature rupture of membranes, placenta previa, congenital heart disease, and other serious congenital diseases that may affect pregnancy.

### Animal experimental design

Eight to ten-week-old, specific-pathogen-free C57BL/6 pregnant mice were purchased by Chengdu Dashuo company. Before and during the experiments, mice were maintained under constant temperature (18–21 °C) and humidity (65–70%) controlled conditions, with a 12:12 h light/dark cycle and free access to food and water. Animal production and usage licenses are SYXK (Yu) 2018–0003 and SCXK (Chuan) 2020–030, respectively. Mice were both drug and test negative and not involved in previous procedures. The pregnant mice were killed by cervical dislocation after the study.

According to the previous method [17, 18], 15 pregnant C57 mice were randomly divided into 5 groups with 3 mice in each group (Table 1). Briefly, the ICP model was established by subcutaneous injection of 17α-ethinyl estradiol (EE, Sigma company, USA). Resveratrol (RES, Sigma Company, USA) and Ursodeoxycholic acid (UDCA, Sigma company, USA) were employed for ICP intervention. LY294002 (MCE, USA), a PI3K inhibitor was used to test the involvement of PI3K/Akt signaling during ICP intervention. In order to observe the distribution of endotoxin in ICP mice, we performed fluorescence imaging of pregnant mice by a small animal in vivo imaging system.To analyze the serum TBA and ALT levels in ICP mice, 0.5 ml of blood was taken from the heart post fetus delivery.The distribution of LPS in the maternal and fetal organs of ICP mice was evaluated by histological analysis.

**Table 1:** Group and treatment of experimental animals

Group	From the 15th day to the 17th day of pregnancy, continuous treatment for 3 days
Group A (Control group)	Subcutaneous injection of vegetable oil, and 0.9% sodium chloride solution was orally given.
Group B (ICP group)	20 mg/(kg·d)EE was subcutaneously injected, and 0.9% sodium chloride solution was orally given.
Group C (ICP + UDCA group)	20 mg/(kg·d)EE was subcutaneously injected, and 62.5 mg/(kg d) Ursodeoxycholic acid was orally given.
Group D (ICP + RES group)	20 mg/(kg·d)EE was subcutaneously injected, and 120 mg/(kg·d) Resveratrol was orally given.
Group E (ICP + RES + LY group)	20 mg/(kg·d)EE was subcutaneously injected, and 120 mg/(kg·d) Resveratrol + 60 mg/(kg·d)LY294002 was orally given.

*EE* 17α- ethinyl estradiol, *LY294002* PI3K inhibitor

### In vivo fluorescence imaging

In order to observe the distribution of endotoxin in pregnant mice, 20 mg/kg fluorescein isothiocyanate labeled LPS (FITC-LPS, Sigma Company, USA) [19] was given on the 18th day and performed fluorescence imaging through the small animal in vivo imaging system. The pregnant mice in each group were given FITC-LPS on the 18th day of pregnancy, and fluorescence imaging was performed on the in vivo fluorescence imaging system (LB983, Berto, Germany) after intragastric administration 6–7 h.

### Histologic analysis

The liver of pregnant mice was fixed in 4% paraformaldehyde, and was cut into paraffin sections and stained with hematoxylin-eosin (HE). The pathological changes in the liver were observed under a light microscope. To detect the fluorescence intensity in different tissues of maternal and fetal mice, Ex495.3 nm and Em516.7 nm were used for fluorescence intensity detection on a spectrophotometer [20]. The standard curve of FITC-LPS was established by dissolving FITC-LPS in normal saline (0.125, 0.25, 0.5, 1, 2, 4, 5μg/ml), and measured on a fluorescence spectrophotometer. After the cesarean section, 100 mg of the liver, mother duodenum, placenta, fetal brain and fetal liver were taken and added with normal saline to make 10% homogenate, followed by centrifugation at 13,000 rpm for 10 min. 0.5 ml of supernatant was dissolved in 0.5 ml of normal saline and 2 ml of anhydrous ethanol for fluorescence measurement.

### Statistical analysis

Graph Pad Prism (version 8.0, GraphPad Software, USA) was used for image processing. Data were expressed as mean ± SEM. Statistical analyses were performed with SPSS software (version 25.0). Independent sample t-test and one-way variance analysis were used to determine statistically significant differences between two and multiple groups. *P* < 0.05 was statistically significant.

## Results

### Analysis of maternal serum LPS and LBP levels in women with ICP

**Fig. 1:**
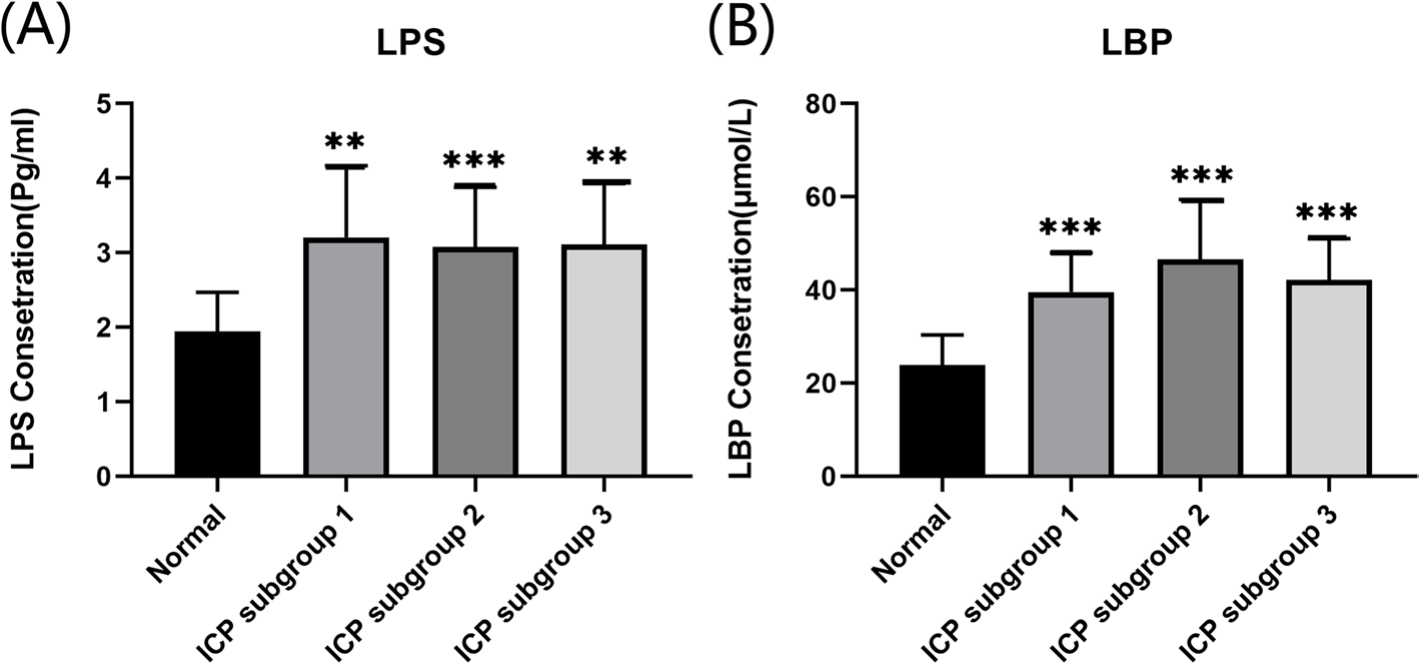
Analyses of serum LPS and LBP levels in ICP women. **A** Serum LPS levels of ICP women. **B** Serum LBP levels of ICP women. Results are shown as mean ± SEM,^**^P < 0.01, ^***^P < 0.001, compared with Normal group

The ELISA kit showed that maternal serum LPS and LBP levels in ICP groups (subgroup 1, subgroup 2, subgroup 3) were significantly higher than that in the normal group (*p* < 0.05) (Fig. 1).

### Histopathological analysis of liver and placental tissues in ICP mice

**Fig. 2:**
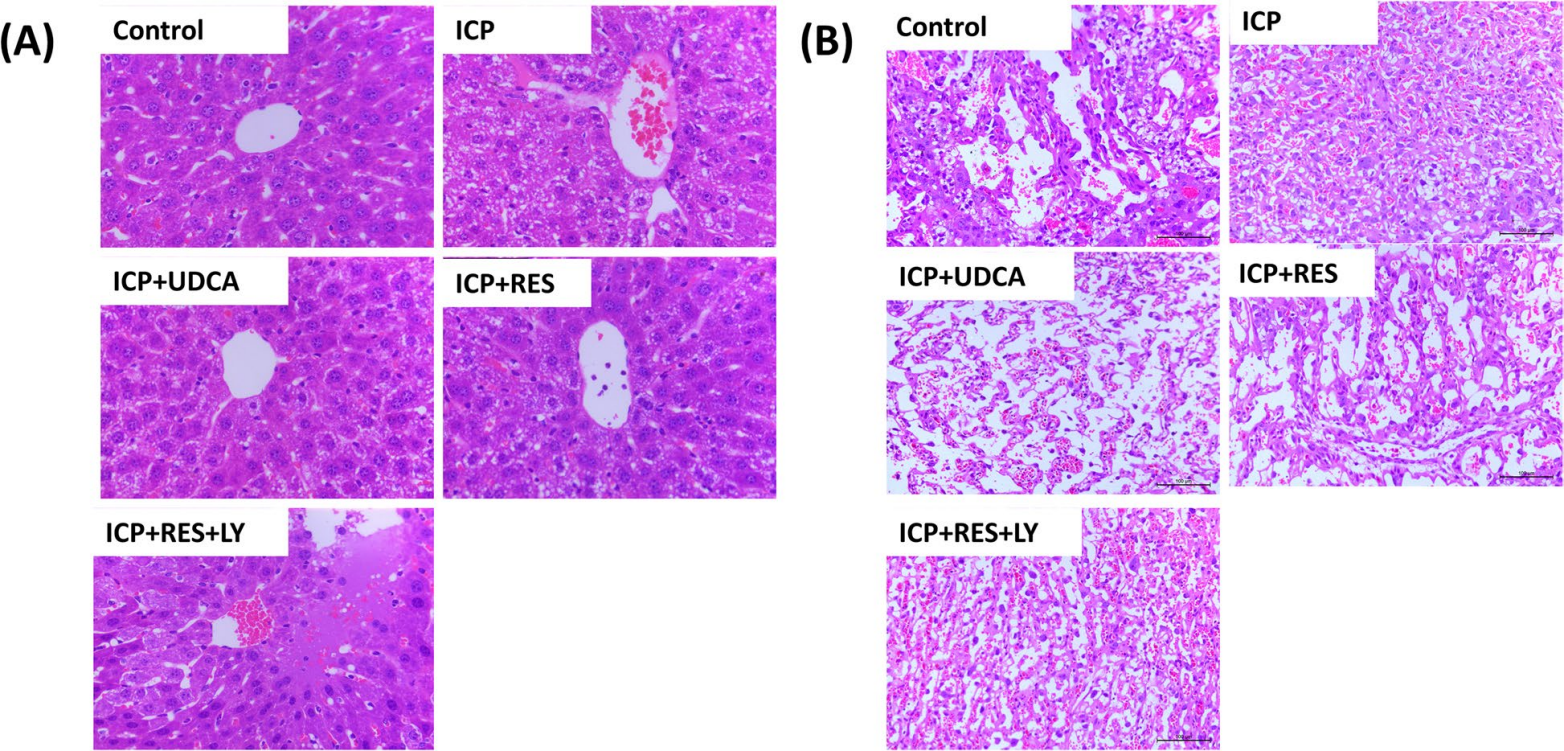
Histopathological images of ICP mice model. **A** The liver tissue of pregnant mice in each group under optical microscope (400X). **B** The placenta tissue of pregnant mice in each group under optical microscope (200X)

As shown in Fig. 2A, HE staining was performed on liver tissue slices of ICP mice. Compared with the control group, the ICP group showed swelling in hepatocytes, water-like degeneration, mesh-like cytoplasm, unclear structure of hepatic sinuses, and severe lesions near the portal area. Compared with the ICP group, the ICP + RES group and ICP + UDCA group partially reversed the swelling in hepatocytes and the mesh-like structure of the cytoplasm. However, the effect of RES was inhibited by LY294002.

As shown in Fig. 2B, compared with the control group, placental villi in the ICP group were obviously edema; villi space was narrow, and bloodshot capillaries increased. Compared with the ICP group, placental villus edema was reduced and villus space was enlarged in ICP + RES and ICP + UDCA groups. Similarly, LY294002 blocked the effect of RES on placental tissue in ICP mice.

### Analyses of serum TBA and ALT levels in ICP mice

The result in mice showed that the TBA level in the ICP group was significantly higher than that in the control group (*P* < 0.05). The TBA levels in the ICP + UDCA and ICP + RES groups were significantly lower than those in the ICP group (*P* < 0.05), while the TBA levels in the ICP + RES + LY group were significantly higher than those in the ICP + RES group (*P* < 0.05). There is no significant difference in TBA level between ICP + UDCA and ICP + RES groups (*P* > 0.05) (Fig. 4A).

The result showed that the ALT level in the ICP group was higher than that in the control group (*P* < 0.05). There is no significant difference in ALT between ICP + UDCA, ICP + RES, ICP + RES + LY, and ICP group (P > 0.05) (Fig. 4B).

### Distribution of LPS in the maternal and fetal organs of ICP mice model

**Fig. 3:**
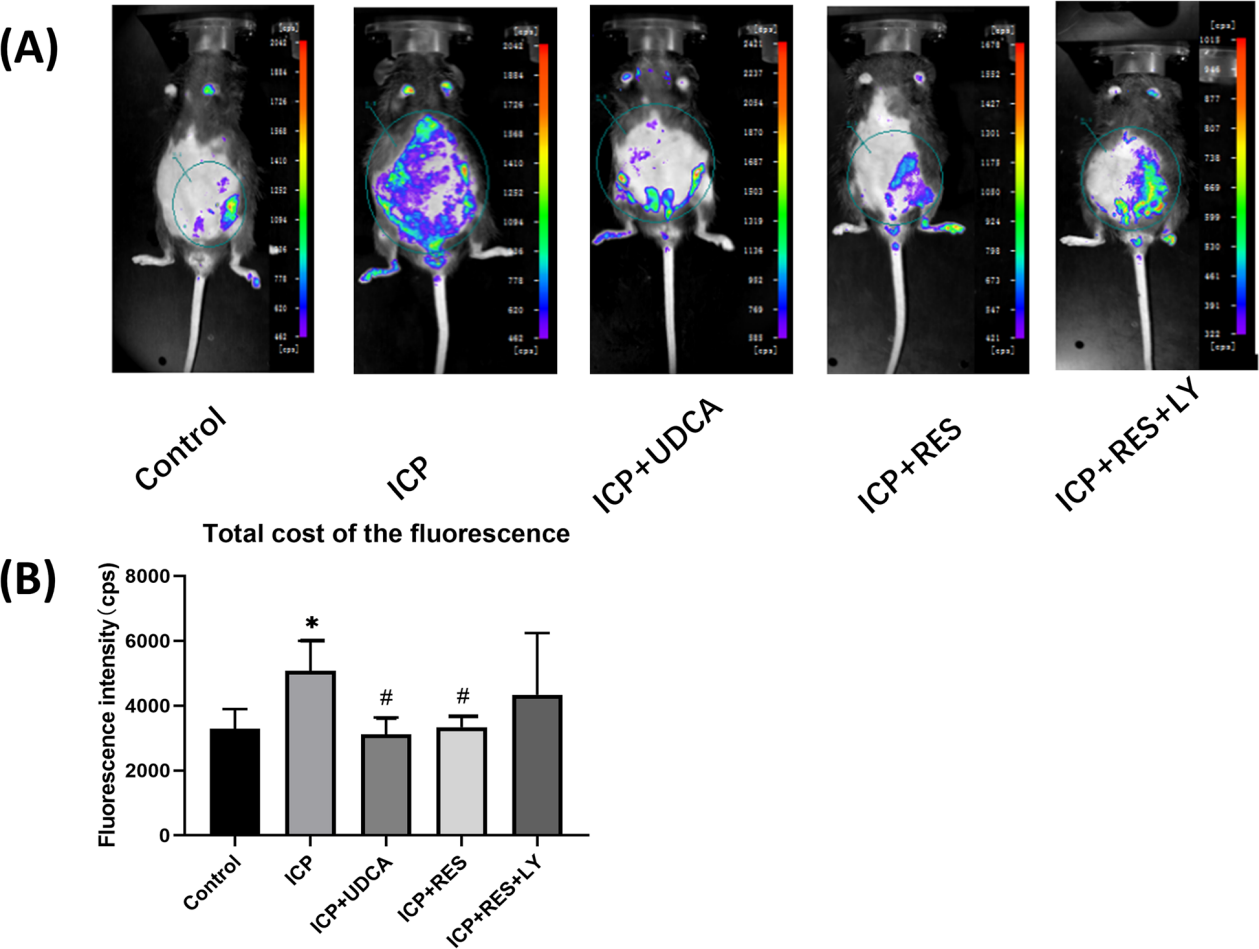
Distribution of LPS in ICP mice. **A** In vivo fluorescence imaging. **B** Quantitative results of fluorescence intensity in abdominal area of ICP mice. Results are shown as mean ± SEM, ^*^P < 0.05, compared with the Control group; ^#^*P* < 0.05, compared with ICP group

As shown in Fig. 3A, fluorescence intensity was detected in the abdominal area of pregnant mice. As shown in Fig. 3B, quantitative results showed that the fluorescence intensity of LPS in the ICP group was significantly higher than that in the control group (*P* < 0.05). The fluorescence intensity of LPS in the ICP + UDCA and ICP + RES groups was significantly lower than that in the ICP group (*P* < 0.05). No significant difference between the ICP + UDCA and ICP + RES groups was noticed.

**Fig. 4:**
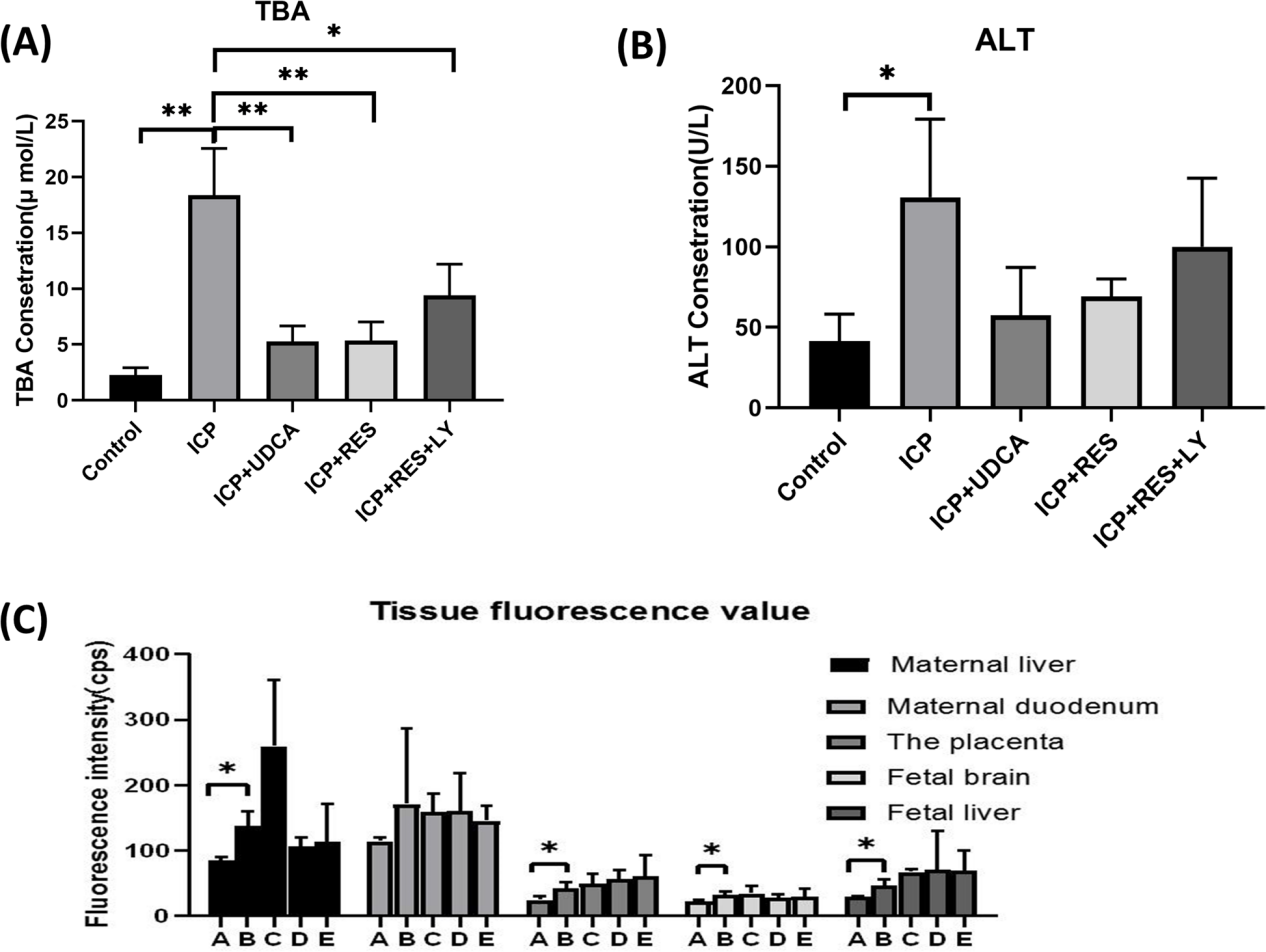
Analyses of serum and fluorescence value of organs. **A** Analyses of serum TBA in ICP pregnant mice (*n* = 3). **B** Analyses of serum ALT in ICP pregnant mice (*n* = 3). **C** Fluorescence value of organs of maternal and fetal mice. (A:Control group; B:ICP group; C:ICP + UDCA group; D:ICP + RES group; E:ICP + RES + LY group). Results are shown as mean ± SEM,*P < 0.05

According to the results detected by the fluorescence spectrophotometer, the fluorescence intensity of the maternal liver, placenta, fetal brain and fetal liver in the ICP group was significantly higher than that in the control group (*p* < 0.05). There was no significant difference between ICP + UDCA, ICP + RES group, and ICP group (*p* > 0.05) (Fig. 4C). The results indicated that LPS levels in the maternal liver, placenta, fetal brain and fetal liver in the ICP group were significantly higher than those in the control group. However, LPS levels in corresponding tissues did not decrease after the UDCA and RES treatment.

### Comparison of stillbirth rate of ICP mice

**Fig. 5:**
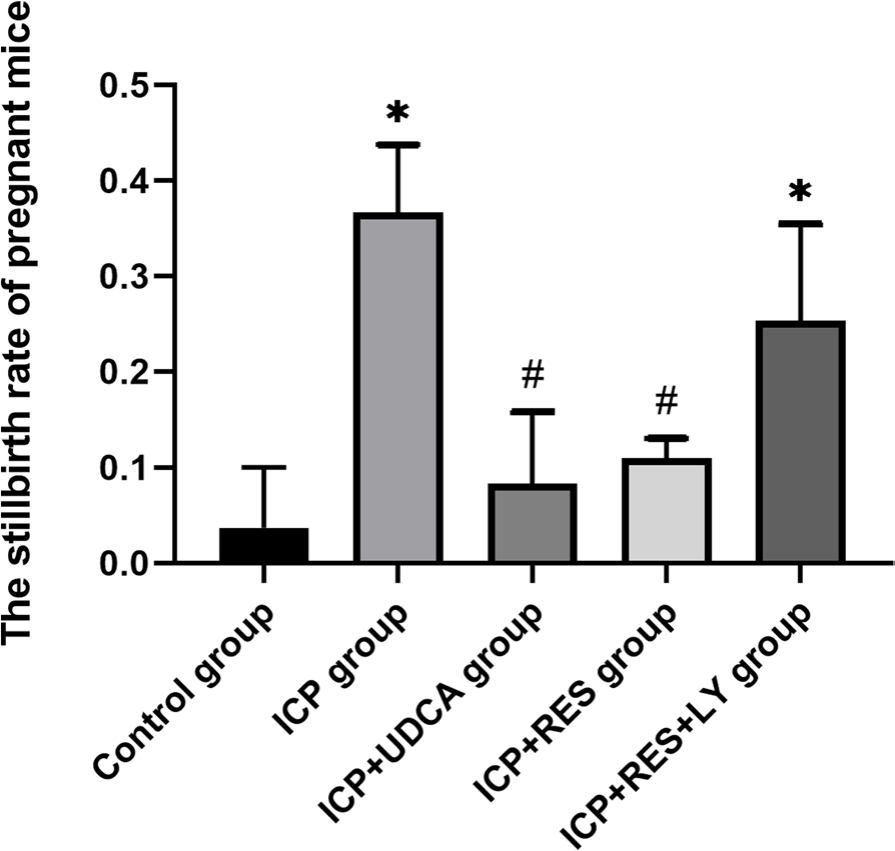
Comparison of stillbirth rate of pregnant mice (*n* = 3). Results are shown as mean ± SEM,* *P* < 0.05, compared with Control group; ^#^*P* < 0.05, compared with ICP group

The stillbirth rate of the ICP group was significantly higher than that of the control group (*P* < 0.05). The stillbirth rate of the ICP + UDCA and ICP + RES groups was lower than that of the ICP group (*P* < 0.05). No significant difference between the ICP + UDCA and ICP + RES groups was noticed (*P* > 0.05) (Fig. 5). The data suggested that UDCA and RES effectively treat ICP, whereas this effect was inhibited by LY294002.

## Discussion

In the present study, we found significantly higher levels of LPS in the serum of women with ICP, and in the serum of the ICP mice. RES and UDCA treatment significantly reduced the distribution of LPS and decreased the stillbirth rate in the ICP mice. Our results indicated a clear association between LPS levels and adverse perinatal outcomes, and the potential application of RES in ICP.

In order to study the distribution of LPS in different tissues of ICP mother and fetal mice, the fluorescence intensity in maternal and fetal mice was detected. Our data show that liver and duodenum are the organs with the greatest accumulation of endotoxin in pregnant mice. In addition, the results also indicated that the fluorescence intensity of endotoxin in the placenta, fetal brain and fetal liver was significantly higher than in the control group. The liver and placenta of ICP pregnant mice showed obvious pathological changes, which may be related to the increase in TBA level and LPS level. This was consistent with previous studies that showed that serum levels of LPS and LBP in the ICP group were significantly higher than those in the control group [8]. In this study, we showed that the fluorescence intensity of LPS in pregnant mice and fetuses decreased significantly post UDCA and RES treatment in ICP mice. Ovadia et al. [21] found that UDCA can reduce the synthesis of bile acid and increase the secretion of bile acid. UDCA can also change the composition of the intestinal flora of ICP women, increase the proportion of bacteroides, and improve intestinal flora disorders in ICP women. The results suggest that the accumulation of endotoxin in different tissues of ICP mice may be related to the imbalance of intestinal flora and the dysfunction of intestinal permeability. Liao et al. [22–24] have been found that RES can activate PI3K / Akt pathway, block NF-kB signaling pathway and improve inflammatory response. Chen et al. [25] found that LY294002 could specifically inhibit the PI3K/Akt pathway and the anti-inflammatory effect of RES. Consistent with previous results, our data also suggested the critical role of the PI3K/Akt pathway in regulating LPS levels during ICP progression.

UDCA is the first-line drug for the treatment of ICP [15]. Glantz et al. [26, 27] showed that UDCA effectively reduced the serum levels of TBA and ALT in ICP women. However, it did not significantly reduce the risk of adverse fetal outcomes (abortion, stillbirth, preterm birth, low birthweight infants, macrosomia, congenital abnormalities, stillbirth and neonatal death). The exact mechanism remained unclear. The present study showed the levels of TBA and ALT in ICP mice were significantly decreased after UDCA treatment, but the fluorescence intensity of LPS in the organs of mice (maternal liver, maternal duodenum, placenta, fetal mice, fetal brain and fetal liver) did not significantly decrease. These data suggested tissue-specific aggregation of LPS in ICP mice. Romero et al. [28] showed that LPS stimulated cytokine and chemokine release, promoted leukocyte infiltration in uterus, placenta and fetal tissue, and syntheses of proinflammatory factors such as prostaglandin, an endogenous cannabinoid, nitrogen and reactive oxygen species (ROS) and metalloproteinases, which are associated with preterm birth in both animal models and humans [29]. Xu et al. [30–32] showed that maternal exposure to LPS will lead to elevated levels of TNF-α in the amniotic fluid and placenta, and may result in intra-uterine fetal death (IUFD), fetal absorption, fetal growth retardation. Izvolskala et al. [33–35] found that LPS-induced cytokine levels affected fetal brain development, and affected long-term neuroendocrine function and fetal behavior. Therefore, it is of considerable significance to explore novel drugs to reduce maternal and fetal LPS levels in order to improve the adverse pregnancy outcomes of ICP. According to the present study, in addition to UDCA, our results showed that RES significantly reduced the level of LPS, and inflammatory tissue reaction in ICP mice. Therefore, this study provided an experimental basis for RES as a new drug to treat ICP in the future.

## Conclusion

The present study provided evidence of endotoxin distribution in maternal liver, placenta, fetal liver, and fetal brain in the ICP mice model. UDCA reduced the serum levels of TBA and ALT in ICP mice, but not the level of LPS in maternal and fetal tissues. RES reduced the levels of TBA and ALT in mice with ICP, and the inflammation caused by LPS. In addition, our data also implies the association between circulating LPS levels and adverse perinatal outcomes. RES might be a potential therapeutic drug for ICP.

## Supplementary Information

**Additional file 1.**

## Data Availability

All data generated or analysed during this study are included in this published article and its supplementary information files.

## Abbreviations

ICP: Intrahepatic cholestasis of pregnancy
LPS: Lipopolysaccharide
LBP: Endotoxin binding protein
UDCA: Ursodeoxycholic acid
RES: Resveratrol
SIRT1: Silent information regulator1
PI3K: Phosphatidylinositol-3-kinase
NF-KB: Nuclear factor-kappa B
LY: LY294002
FITC-LPS: Fluorescein isothiocyanate labelled LPS
TBA: Total bile acid
ALT: Alanine aminotransferase
EE: 17α-ethinyl estradiol
DMSO: Dimethyl sulfoxide
T2D: Type 2 diabetes
IBD: Inflammatory bowel disease
TLR4: Toll like receptor 4
NO: Nitric oxide
TNF-α: Tumor necrosis factor α
ROS: Reactive oxygen species
IUFD: Intra-uterine fetal death
HE: Hematoxylin-eosin
