# Supplementary figures and images for: Distribution of endotoxin in maternal and fetal body with intrahepatic cholestasis of pregnancy and its association with adverse fetal outcome
Source: BMC Pregnancy Childbirth. 2022 Dec 8;22:920. doi: 10.1186/s12884-022-05235-4 (PMC9733156; doi:10.1186/s12884-022-05235-4)

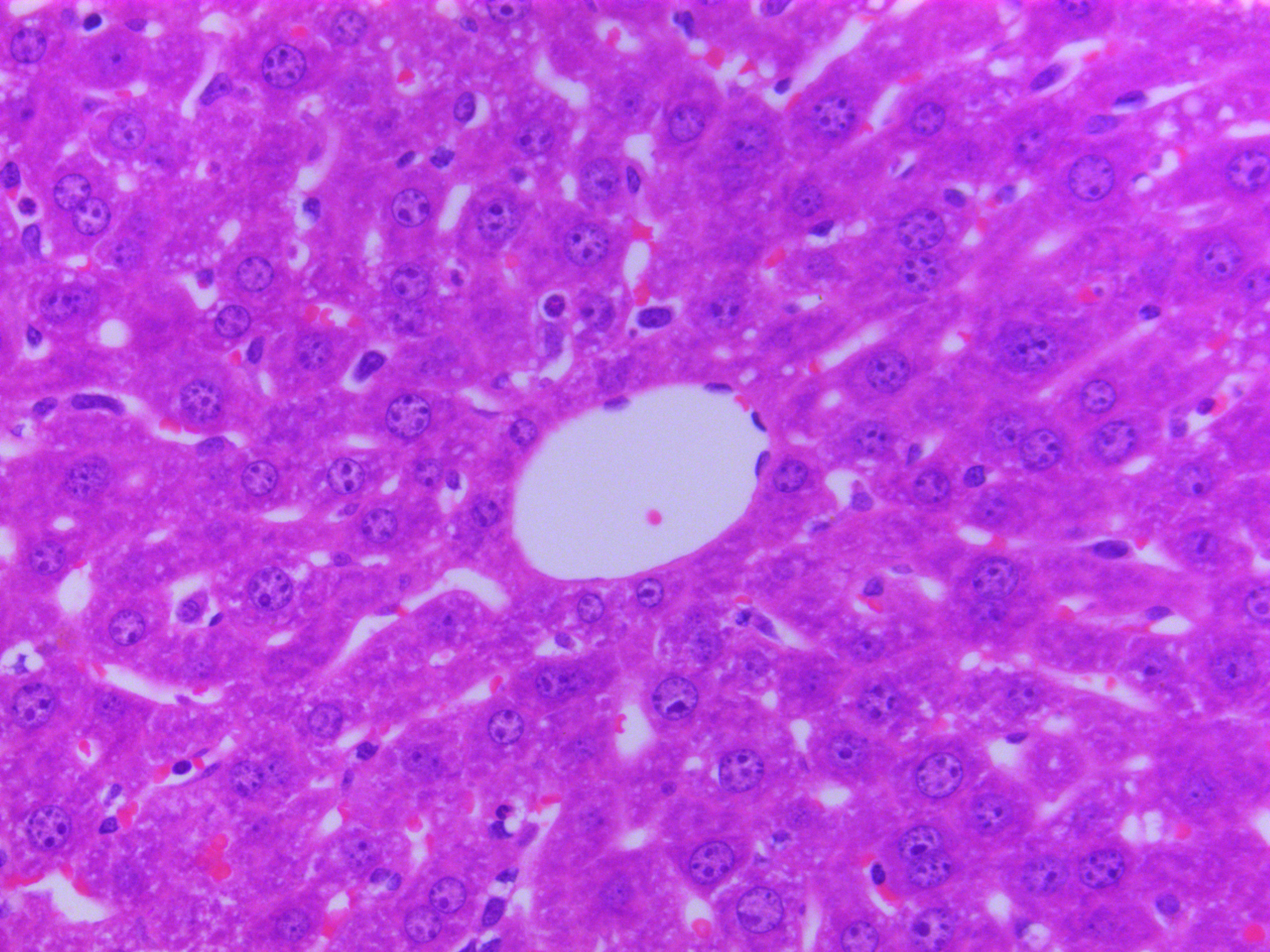

Supplement: Supplementary file 1 — Additional file 1. [file 12884_2022_5235_MOESM1_ESM.zip › FIG.2(A)-ControlR5.tif]

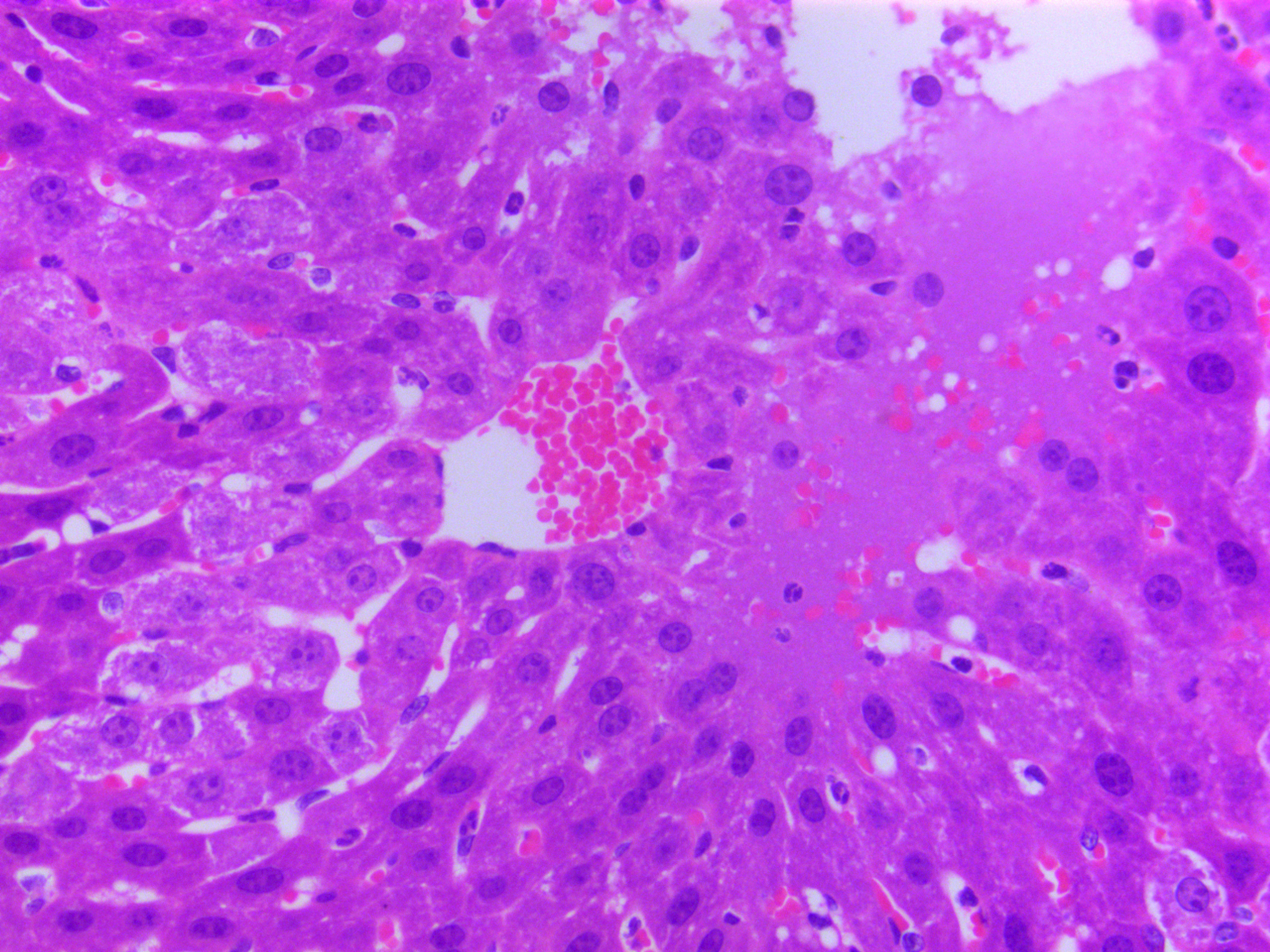

Supplement: Supplementary file 1 — Additional file 1. [file 12884_2022_5235_MOESM1_ESM.zip › FIG.2(A)-ICP+RES+LYR5.tif]

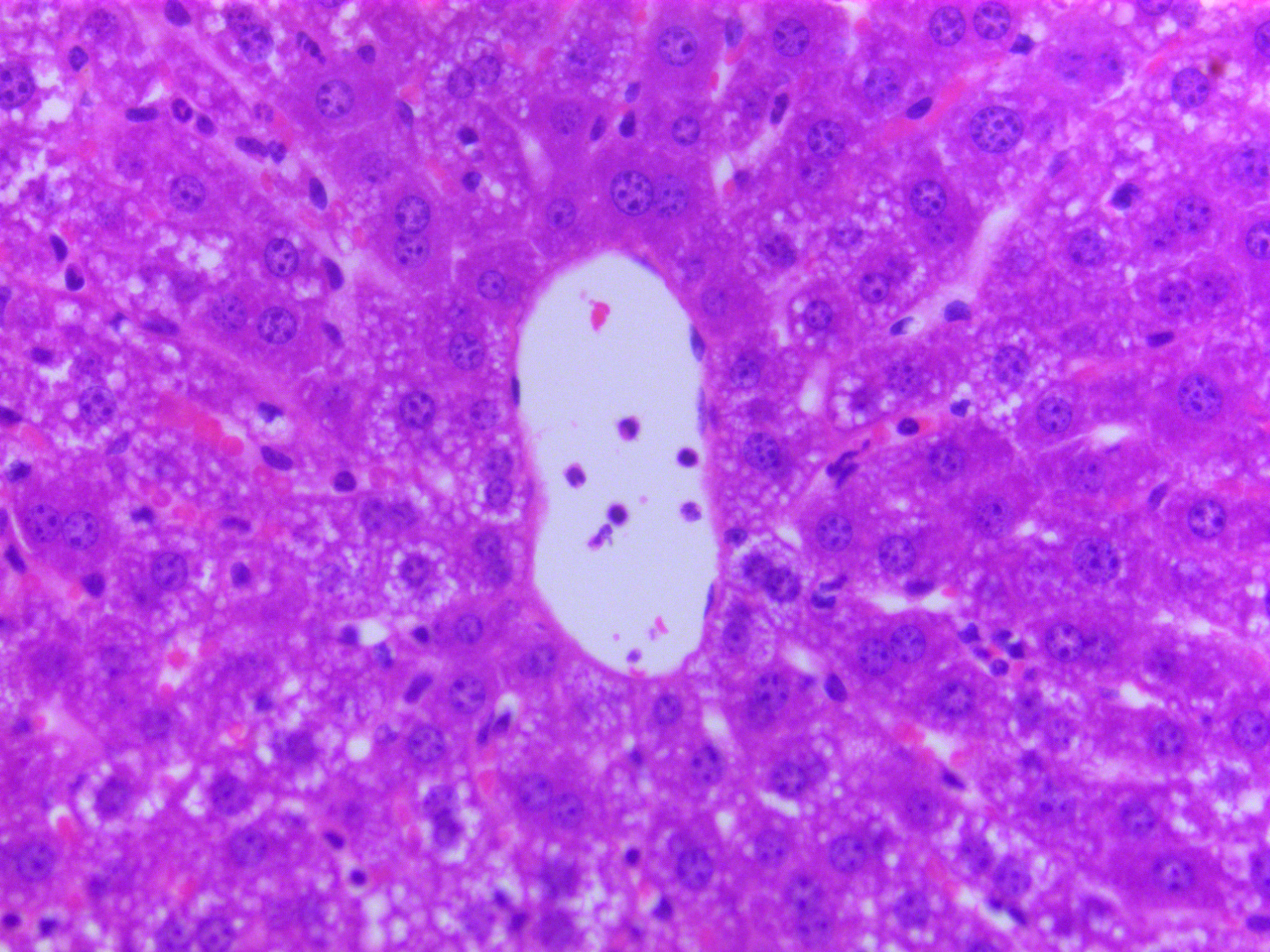

Supplement: Supplementary file 1 — Additional file 1. [file 12884_2022_5235_MOESM1_ESM.zip › FIG.2(A)-ICP+RESR5.tif]

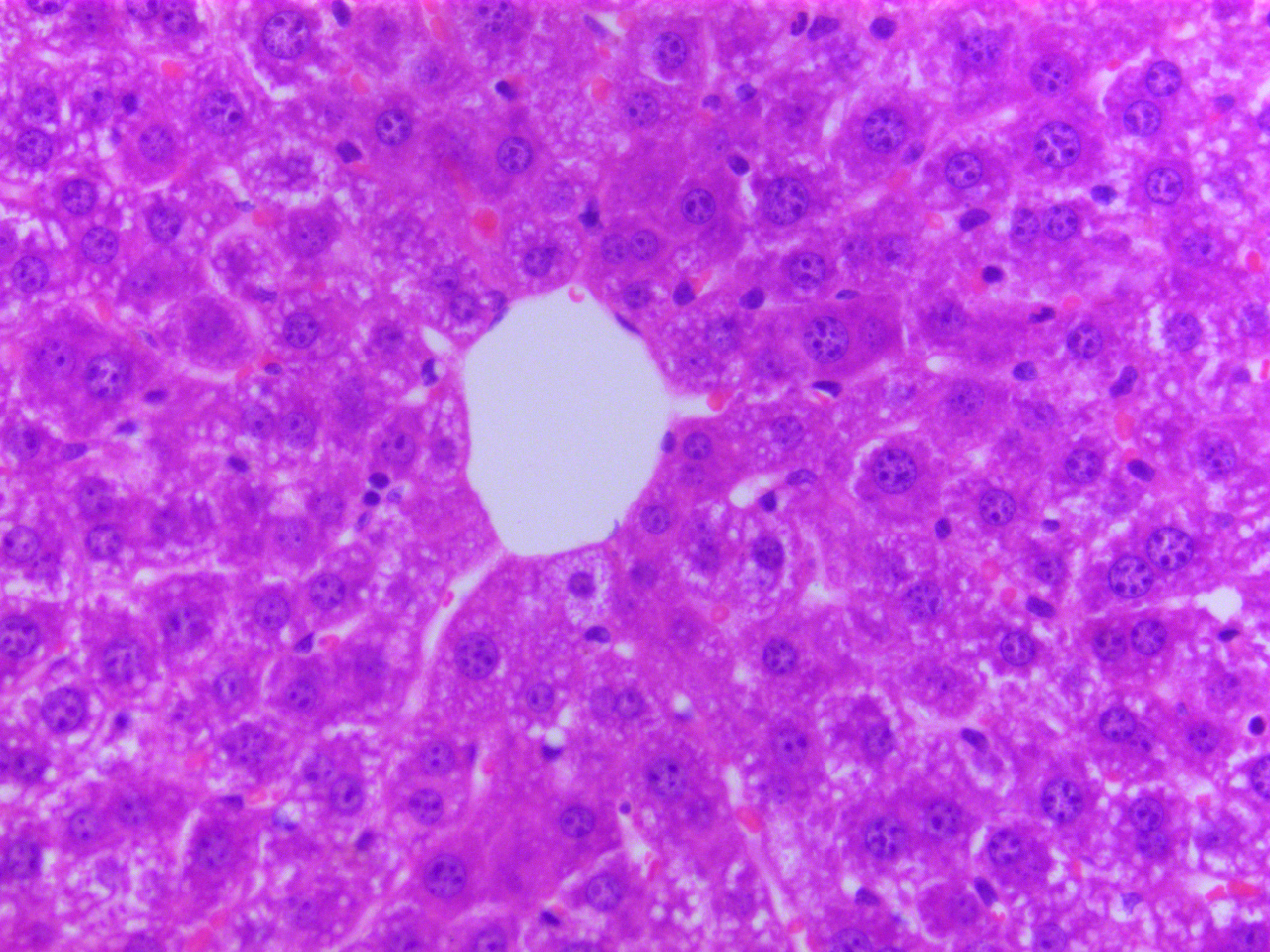

Supplement: Supplementary file 1 — Additional file 1. [file 12884_2022_5235_MOESM1_ESM.zip › FIG.2(A)-ICP+UDCAR5.tif]

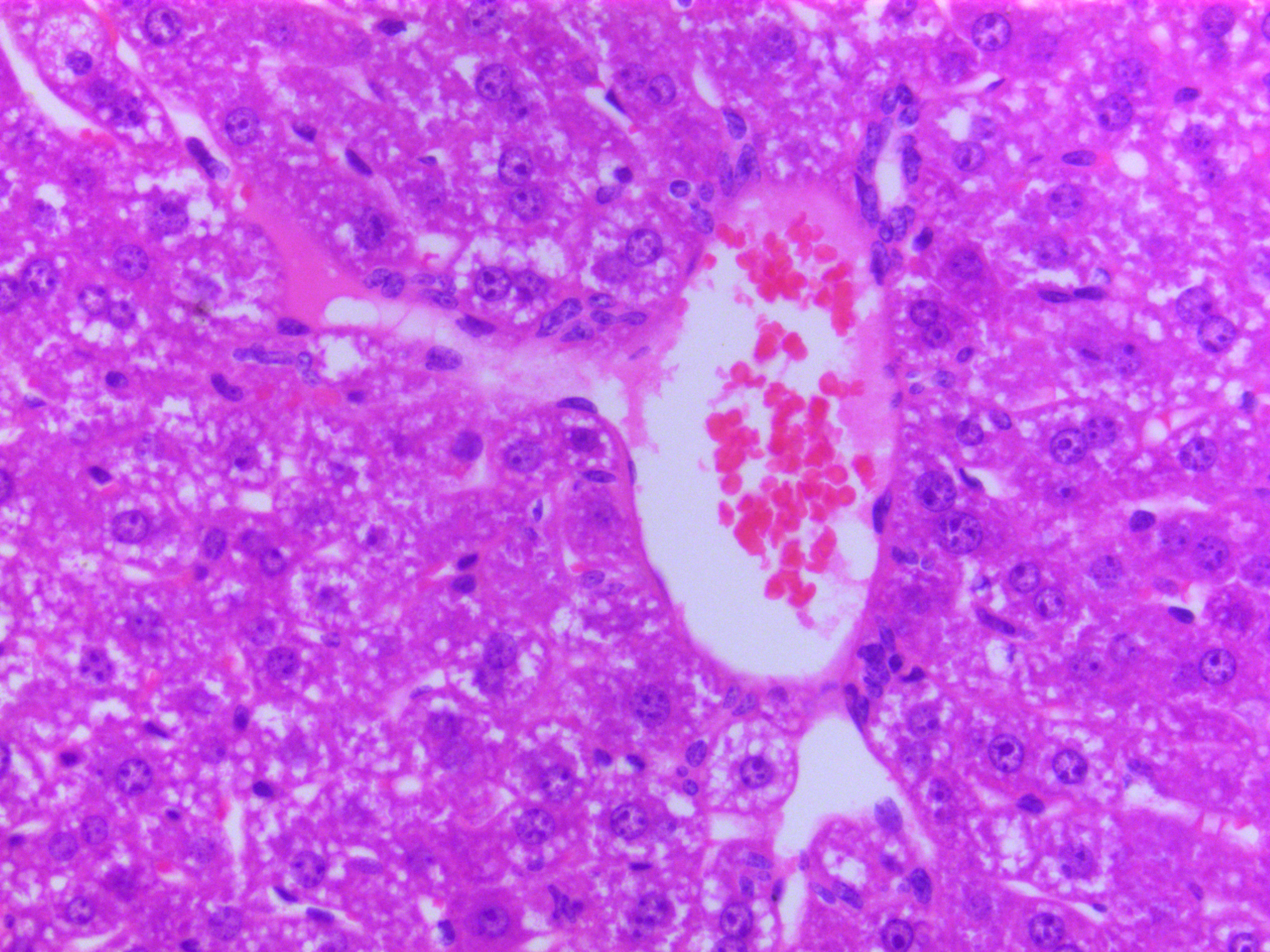

Supplement: Supplementary file 1 — Additional file 1. [file 12884_2022_5235_MOESM1_ESM.zip › FIG.2(A)-ICPR5.tif]

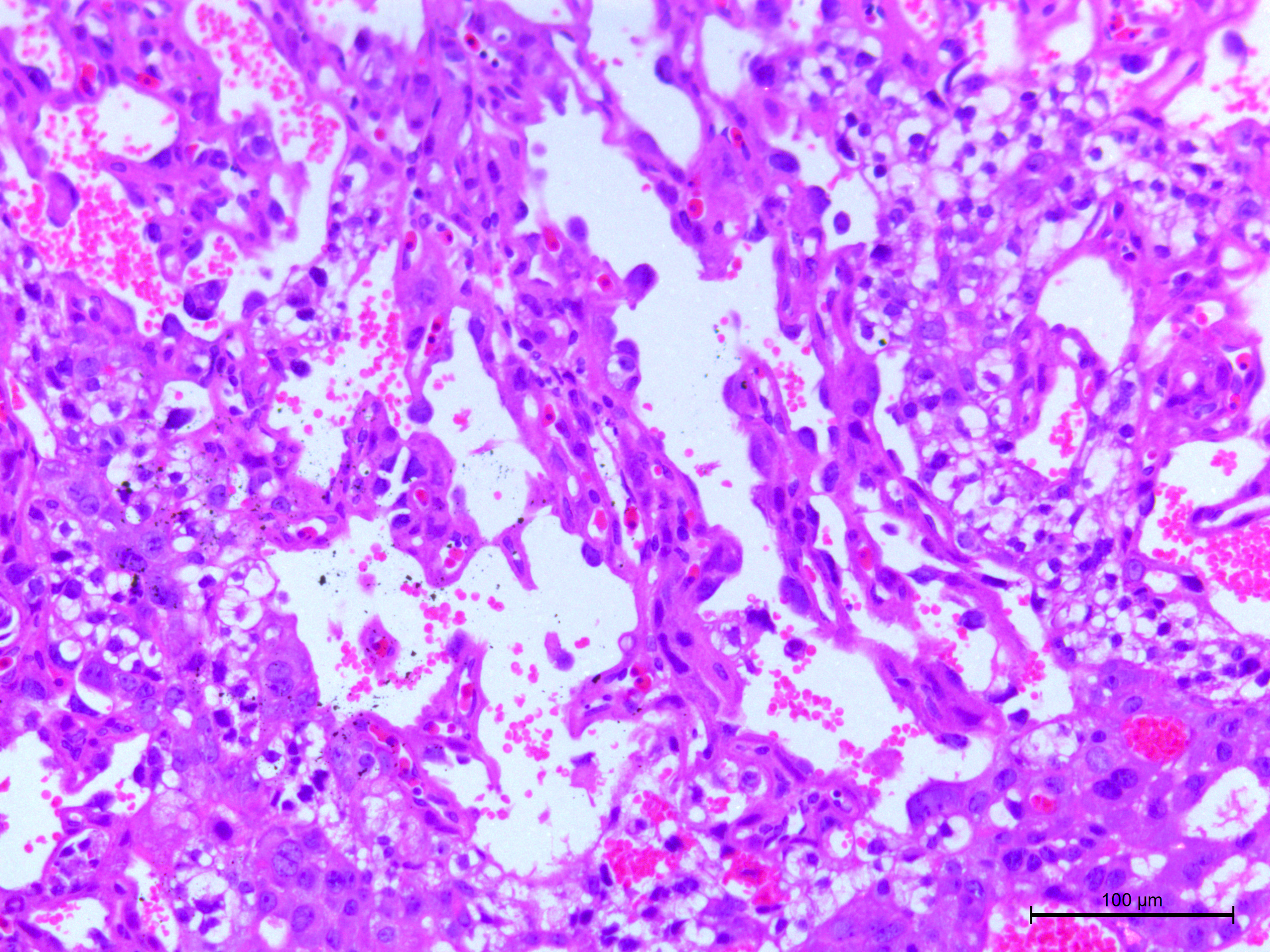

Supplement: Supplementary file 1 — Additional file 1. [file 12884_2022_5235_MOESM1_ESM.zip › FIG.2(B)-ControlR5.tif]

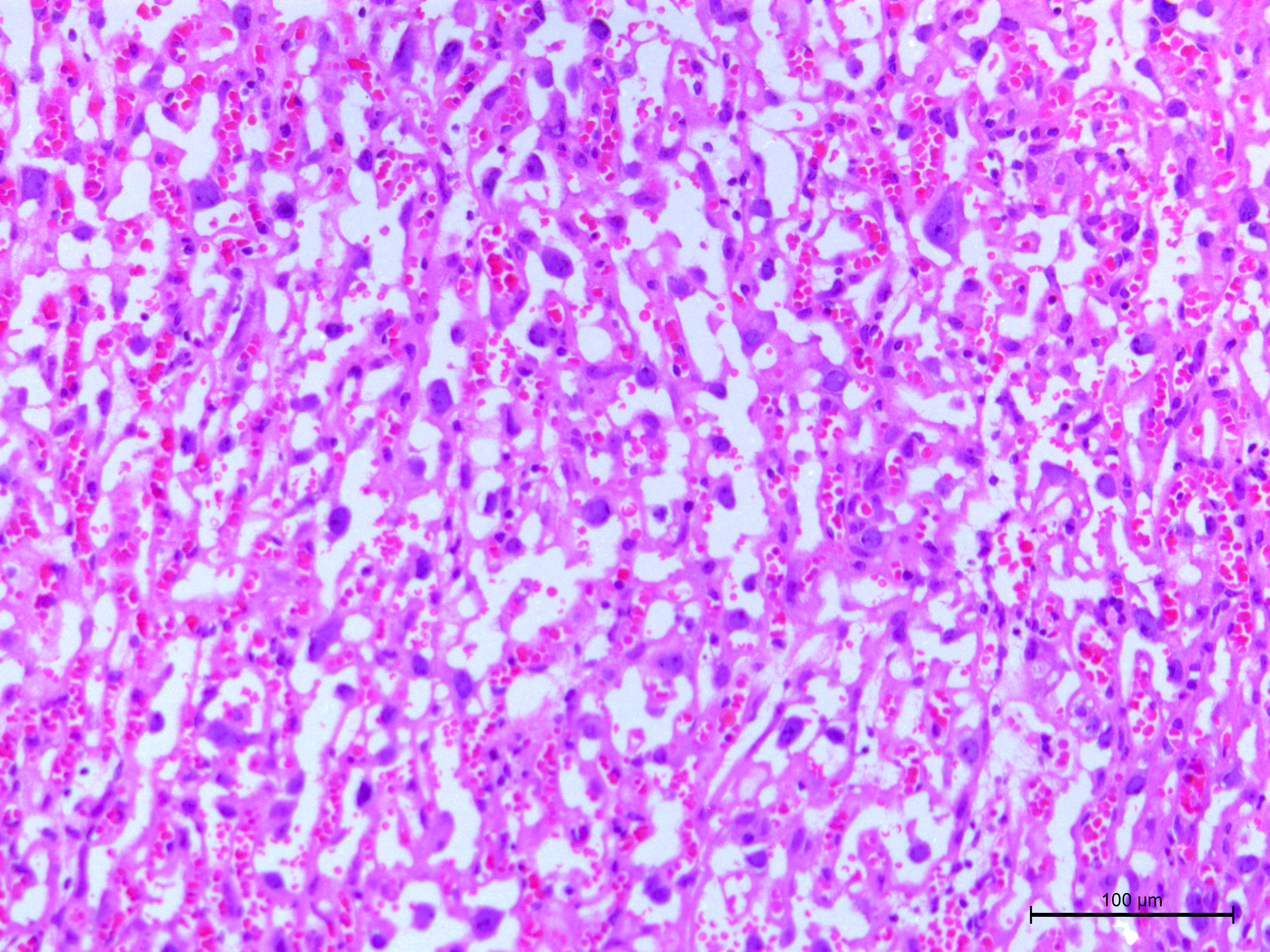

Supplement: Supplementary file 1 — Additional file 1. [file 12884_2022_5235_MOESM1_ESM.zip › FIG.2(B)-ICP+RES+LYR5.tif]

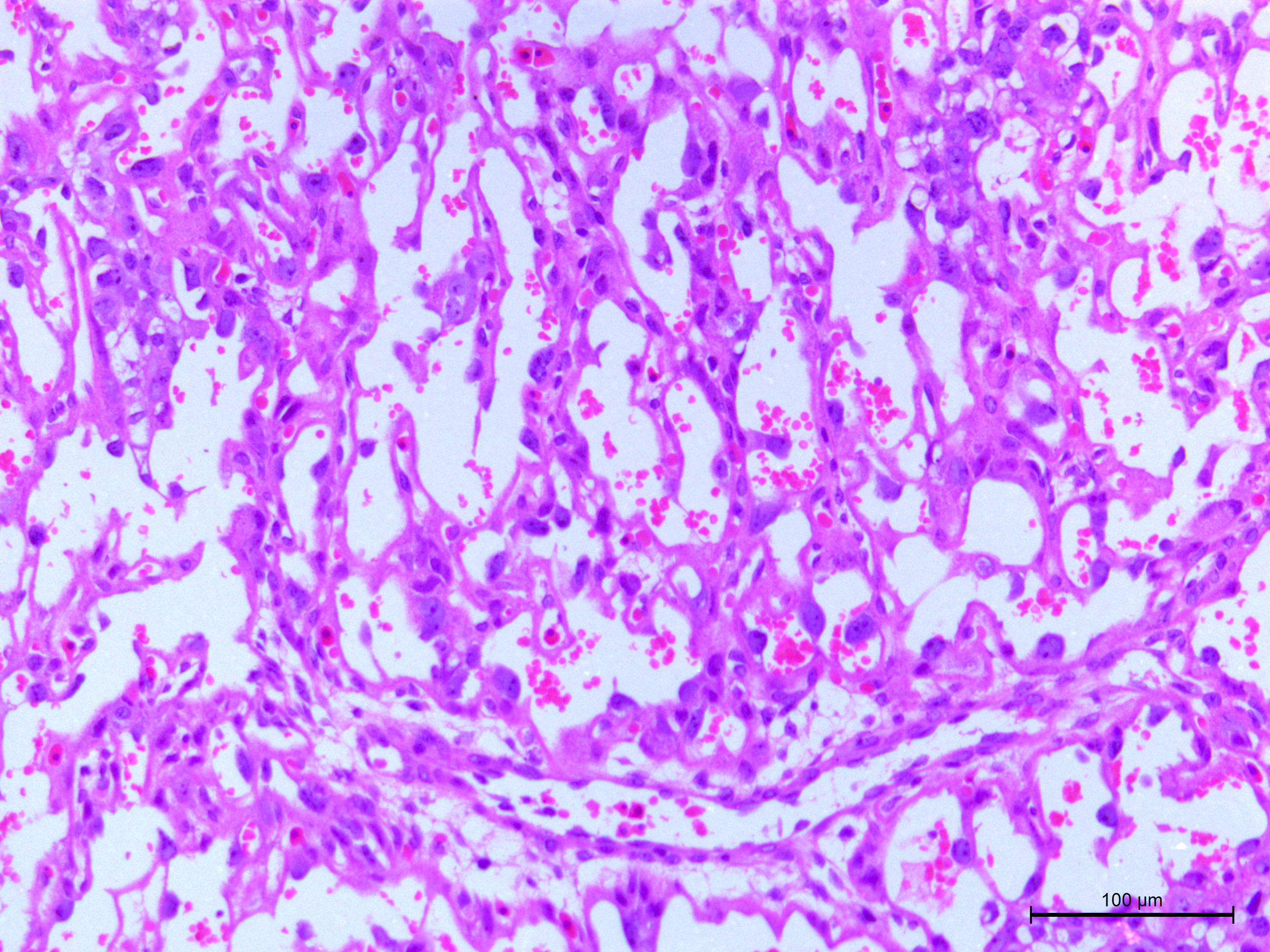

Supplement: Supplementary file 1 — Additional file 1. [file 12884_2022_5235_MOESM1_ESM.zip › FIG.2(B)-ICP+RESR5.tif]

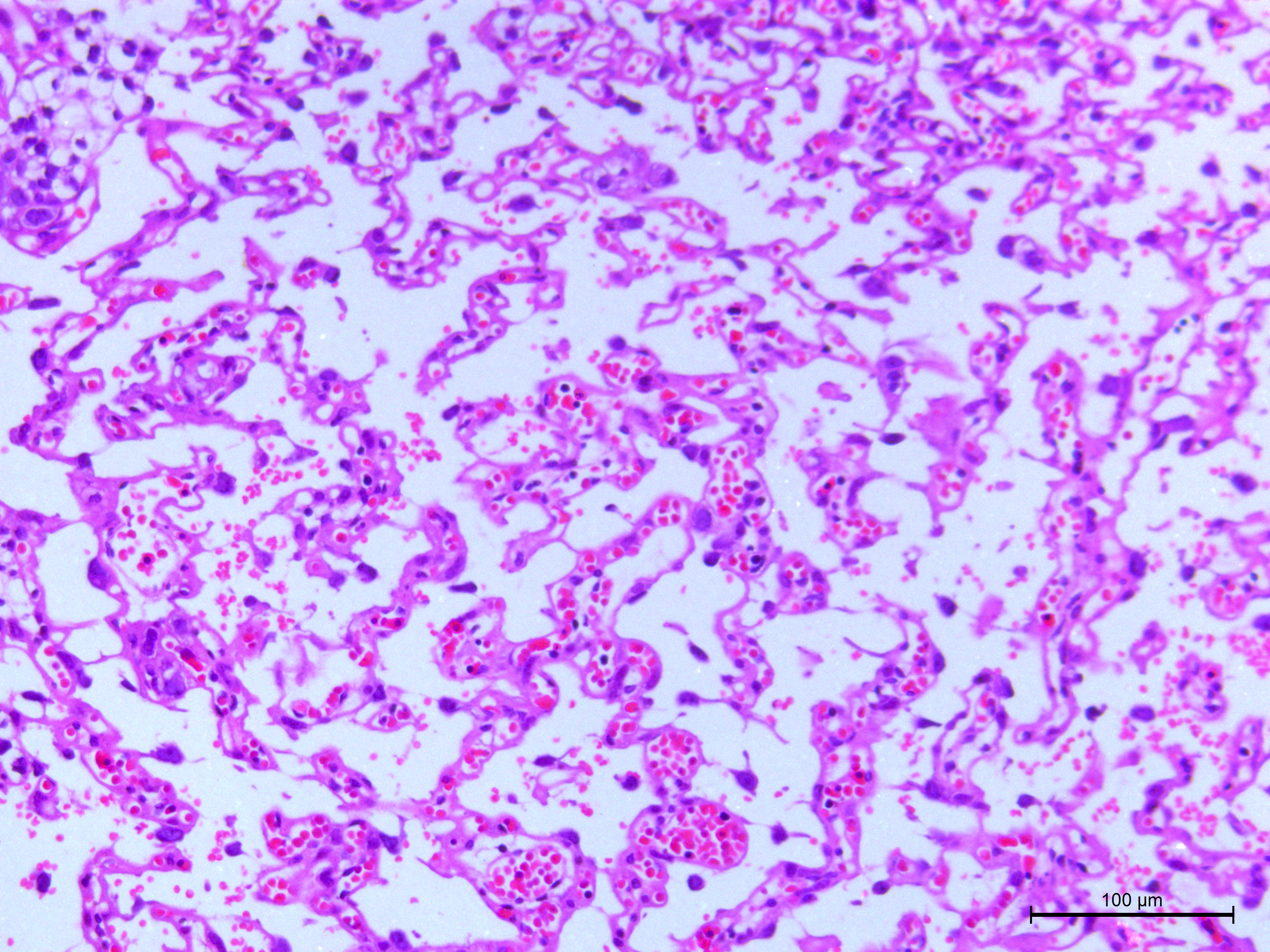

Supplement: Supplementary file 1 — Additional file 1. [file 12884_2022_5235_MOESM1_ESM.zip › FIG.2(B)-ICP+UDCAR5.tif]

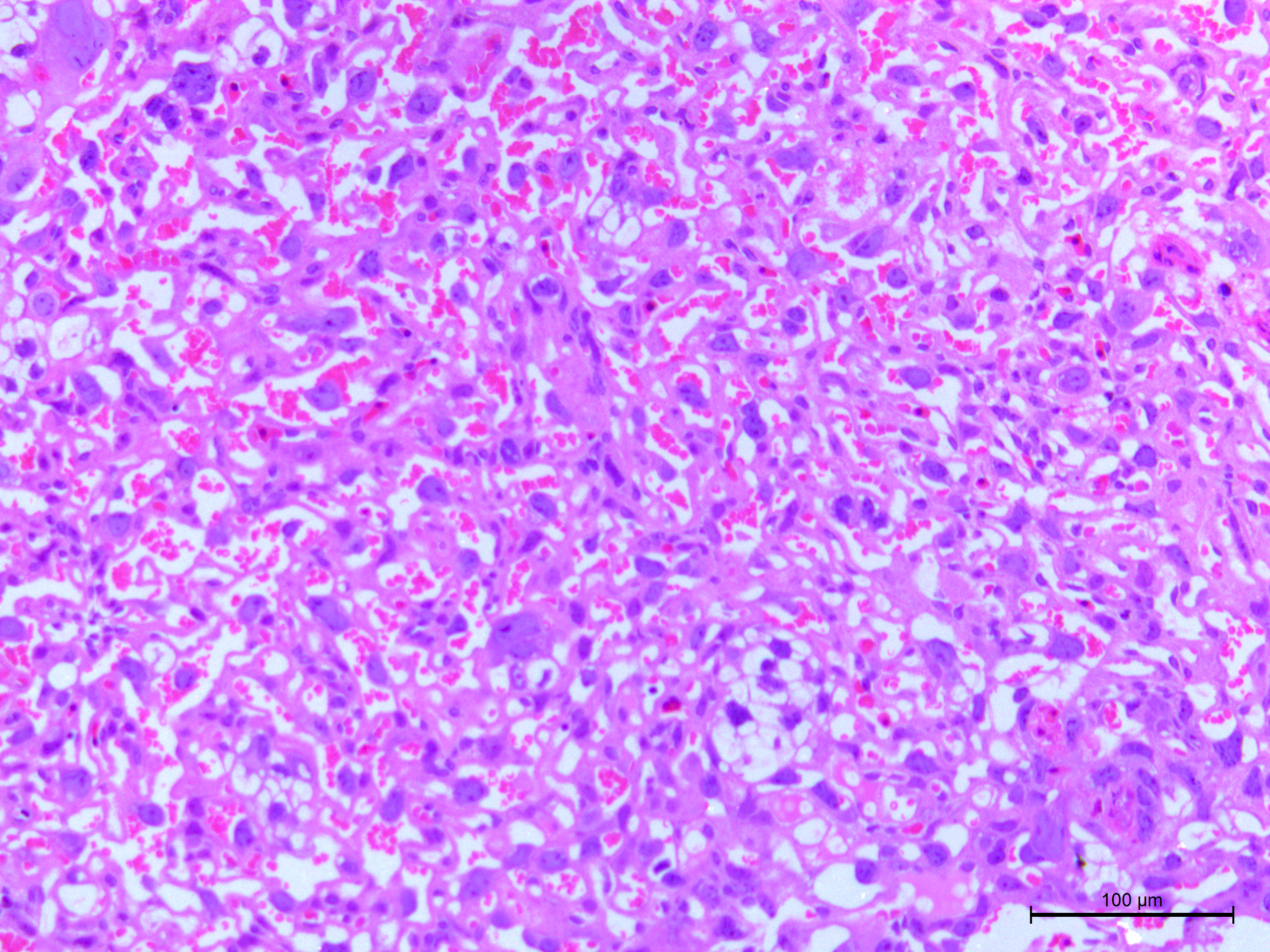

Supplement: Supplementary file 1 — Additional file 1. [file 12884_2022_5235_MOESM1_ESM.zip › FIG.2(B)-ICPR5.tif]

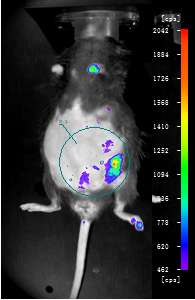

Supplement: Supplementary file 1 — Additional file 1. [file 12884_2022_5235_MOESM1_ESM.zip › FIG.3(A)-ControlR5.png]

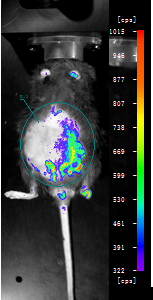

Supplement: Supplementary file 1 — Additional file 1. [file 12884_2022_5235_MOESM1_ESM.zip › FIG.3(A)-ICP+RES+LYR5.png]

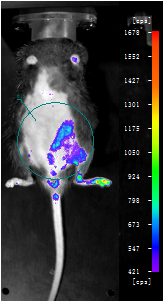

Supplement: Supplementary file 1 — Additional file 1. [file 12884_2022_5235_MOESM1_ESM.zip › FIG.3(A)-ICP+RESR5.png]

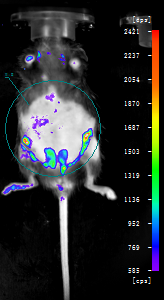

Supplement: Supplementary file 1 — Additional file 1. [file 12884_2022_5235_MOESM1_ESM.zip › FIG.3(A)-ICP+UDCAR5.png]

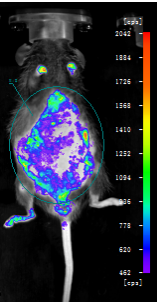

Supplement: Supplementary file 1 — Additional file 1. [file 12884_2022_5235_MOESM1_ESM.zip › FIG.3(A)-ICPR5.png]
